# Cannabis for morning sickness: areas for intervention to decrease cannabis consumption during pregnancy

**DOI:** 10.1186/s42238-023-00184-x

**Published:** 2023-06-17

**Authors:** Karli Swenson

**Affiliations:** Department of Pediatrics, Section of Developmental Biology, University of Colorado Anschutz Medical Campus, 12800 E 19th Avenue, RC1 North MS 8313, Aurora, CO 80045 USA

**Keywords:** Pregnancy, Morning sickness, Cannabis, Marijuana, Cannabidiol

## Abstract

**Background:**

Cannabis use during pregnancy is increasing, with 19–22% of patients testing positive at delivery in Colorado and California. Patients report using cannabis to alleviate their nausea and vomiting, anxiety, and pain. However, preclinical and clinical data highlight harmful effects to offspring physiology and behavior following fetal cannabis exposure. This narrative review identifies potential areas for intervention to decrease cannabis consumption during pregnancy.

**Methods:**

A combination of keywords, including “cannabis”, “cannabis”, “weed”, “pregnancy”, “morning sickness”, “child protective services”, and “budtender” were searched in databases such as PubMed and Google Scholar, as well as in social media forums, governmental webpages, and other publicly available sources.

**Results:**

The literature search identified several areas for intervention to reduce cannabis use during pregnancy, including physician and pharmacist training, engagement with pregnant patients, regulation of dispensary workers, and the role of child protective services.

**Discussion:**

This comprehensive review identifies multiple areas for improvement to benefit pregnant patients. Recommendations are independent and can be implemented simultaneously by the identified groups. Limitations of this research includes the relatively limited availability of data focused specifically on cannabis consumption during pregnancy and the complexity of the sociopolitical field of substance use during pregnancy.

**Conclusions:**

Cannabis consumption during pregnancy is increasing and causes harm to the developing fetus. To educate pregnant patients about these risks, we must address the gaps in education from multiple contact points.

## Introduction and background

### Morning sickness is debilitating and pregnant patients need relief

Fifty to ninety percent of pregnant people experience nausea and vomiting during their pregnancy (NVP), and up to 114,000 pregnant people in the USA suffer yearly from hyperemesis gravidarum, which is persistent nausea and vomiting daily throughout pregnancy. Persistent nausea leaves pregnant patients desperate for relief, causing many people to choose cannabis as an anti-emetic (Barbosa-Leiker et al. 2020).

### Cannabis is an effective anti-emetic

Cannabis components, including tetrahydrocannabinol (THC), cannabidiol (CBD), and minor cannabinoids, are under current clinical investigation for the anti-emetic effects (Marijuana and Cancer 2022). Synthetic THC (dronabinol) and a synthetic cannabinoids nabilone are FDA approved for chemotherapy induced nausea and vomiting (Marijuana and Cancer 2022). Nabiximols are cannabinoid based oral sprays currently under investigation for chemotherapy induced nausea (Marijuana and Cancer 2022). Clinical and preclinical data also show CBD as an effective anti-emetic (Parker et al. 2011).

### Cannabis components cross the placenta into the fetal blood stream

Both THC and CBD are small, lipophilic molecules that pass the maternal-placental-fetal pathway into the bloodstream of the fetus (Hutchings et al. 1989). After birth, THC and CBD diffuse through breastmilk into the blood stream of a baby (Ryan et al. 2018).

### Pregnant people are increasingly consuming cannabis

The number of people who consume cannabis is steadily increasing in the USA as a whole and among pregnant people (Brown et al. 2017). Cannabis is the most commonly used federally illicit substance by pregnant people (Jutras-Aswad et al. 2009), closely following consumption of legal recreational substances such as nicotine and alcohol (Jutras-Aswad et al. 2009). The self-reported prevalence of cannabis consumption among pregnant people in the USA falls between 2 and 7% of pregnant people (Brown et al. 2017), (Ko et al. 2020), (Crume et al. 2018), which has increased 62% between 2002 and 2014 (Brown et al. 2017). Because of the potential legal/social implications of reporting substance use to a provider, it is commonly understood that self-report rates are an underestimate of true consumption rates (Metz et al. 2019), (Young-Wolff et al. 2017). In Colorado, birthing patients were 22.5% positive for THC at labor and delivery (Metz et al. 2019). In California, birthing parents 18–24 years old were 19% positive for cannabis use at 8 weeks gestation (Young-Wolff et al. 2017). However, rates of consumption are significantly different between groups of pregnant people based on age, socioeconomic status, location, race, insurance status, and education (Ko et al. 2020). Cannabis consumption rates are highest among young, socioeconomically disadvantaged people (Ko et al. 2020).

### There is little information available about how fetal cannabis exposure affects developing offspring

Studies highlighting the risks of fetal exposure to cannabis are increasing (Chia-Shan et al. 2011), (Viveros and Marco 2015), (Jansson et al. 2018), (Hurd et al. 2019). In human retrospective studies, children exposed to cannabis in-utero are more likely to be admitted to the neonatal intensive care unit, have lower birth weights, and increased preterm delivery (Marchand et al. 2022). Furthermore, retrospective studies show that cannabis exposure during fetal development increases chances of ADHD and anxiety in the child as they reach puberty (Grant et al. 2018).

### Primary goal of this review

The goal of this narrative review is to highlight the complex sociopolitical and regulatory frameworks surrounding cannabis consumption during pregnancy. This review highlights why pregnant patients choose to consume cannabis, identifies multiple hindrances in educating these patients, and proposes multiple specific recommendations for researchers, clinicians, public health professionals, and legislatures to implement to close gaps in knowledge and benefit pregnant patients.

## Methods

This paper is a narrative review which focuses on the sociopolitical, legal, and medical frameworks in which pregnant people navigate to understand the safety or potential harm of consuming cannabis during pregnancy. A combination of keywords, including “marijuana”, “cannabis”, “weed”, “pregnancy”, “morning sickness”, “child protective services”, and “budtender” were searched in databases such as PubMed and Google Scholar. Additionally, organization of this paper is based on the multiple contact points pregnant patients have for obtaining information, including physicians, pharmacists, peers, the internet and social media, dispensary workers, child protective services, and the government. Facets of the research that are publicly available, like pro-cannabis social media pages, and governmental webpages, were found using internet searches for the aforementioned key terms.

## Results

### Cannabis consumption from the view of a pregnant patient

#### Pregnant people consume cannabis to alleviate symptoms of pregnancy

The majority of people who report regular cannabis consumption during pregnancy use cannabis to alleviate symptoms associated with pregnancy (Ko et al. 2020), (Westfall et al. 2009). In two independent studies, pregnant people reported the treatment of nausea and/or vomiting (77.8%, 77%), stress/anxiety (81.5%, 75%), pain (55.1%, 83%), insomnia (74%), and appetite (70%) (Ko et al. 2020), (Westfall et al. 2009). People also report the recreational aspect of cannabis, as 45.7% of respondents states they consume to have fun and/or relax (Ko et al. 2020). In interviews, these patients report fear of the effects of pharmaceuticals on their developing babies and the belief that cannabis was their only safe option (Barbosa-Leiker et al. 2020). People note that they were not haphazardly consuming cannabis or its components and frequently reevaluated their symptoms and need for treatment throughout the pregnancy (Barbosa-Leiker et al. 2020).

When interviewed about their cannabis use during pregnancy, patients told stories of perceived health benefits, fear of physicians, and lack of options to treat their nausea, pain, or anxiety associated with pregnancy (Barbosa-Leiker et al. 2020). Patients described feeling that cannabis was the only way they were able to keep food down and were worried that they could not provide nutrition for their developing babies without help from cannabis (Barbosa-Leiker et al. 2020). They discussed the quality-of-life component when cannabis consumption allowed them to relax, to better care for their existing kids, and to decrease their anxiety (Barbosa-Leiker et al. 2020). When prompted on their reasoning to believe that cannabis consumption was safe for the baby, they explained that because cannabis is a “natural” substance that it must not pose as many risks as pharmaceuticals despite acknowledging risks of tobacco, another natural substance (Chang et al. 2019). These interviewees explained that compared to other drugs that they deemed to be harmful during pregnancy (e.g., methamphetamines, heroin), cannabis must be more safe because it is not a “hard drug” (Chang et al. 2019).

#### Cannabis companies often do not label products with pregnancy and breastfeeding concerns

In legalized states, there are currently no legal restrictions on selling cannabis or any of its component parts to pregnant people, as long as they are of legal age within that state. As of April 2023, 15 jurisdictions currently require, or will require in 2023, some form of cannabis product labeling to include a warning of potential health risks when consumed by pregnant or breastfeeding people, like those found on alcoholic beverages (Cannabis Regulation Fact Sheet 2022). Some states use “do not use if pregnant or breastfeeding,” like Vermont and Alaska, and some like Michigan are more detailed, saying “use by pregnant or breastfeeding women, or by women planning to become pregnant, may result in fetal injury, preterm birth, low birth weight, or developmental problems for the child” (Cannabis Regulation Fact Sheet 2022).

#### Pregnant patients fear potential repercussions about reporting their use of cannabis to physicians

When asked about if they discuss or disclose their cannabis consumption to their physician, the pregnant patients discussed social stigmas, fear of child protective services, and fear of legal repercussions (Barbosa-Leiker et al. 2020). Some patients mentioned the fear of urine drug screens, the judgment from clinicians, and the lack of communicated information (Barbosa-Leiker et al. 2020). Patients discussed their fear of having their new baby or their existing children taken away from them because of their cannabis use (Barbosa-Leiker et al. 2020). However, patients reported contradicting instructions from clinicians about legality (Barbosa-Leiker et al. 2020). Some patients were told that child protective services (CPS) would get involved, and others were told the risk was minimal (Barbosa-Leiker et al. 2020). Some patients had neutral interactions with providers including obstetricians, gynecologists, pediatricians, and midwives, where there providers did not guide the patients to cease nor continue consumption (Barbosa-Leiker et al. 2020), (Chang et al. 2019). However, among people who quit smoking cannabis regularly throughout pregnancy, only 27% of respondents listed a doctor’s recommendation as motivation to quit, while 74% cited avoiding being a “bad example” (Mark et al. 2017). Pregnant people have poor knowledge of the potential risks of cannabis use during pregnancy (Ng et al. 2020). Specifically, more than 90% of pregnant subjects reported they would be more likely to use cannabis during pregnancy if it were fully legalized (Ng et al. 2020).

#### The fear of legal repercussions following gestational cannabis consumption is well founded

CPS is a group of governmental entities in the USA which form community networks with the goal to strengthen families and keep children safe (Child Welfare Information Gateway 2020). These entities include the departments of social services or child and families services, who work alongside private child welfare agencies and community-based organizations (Child Welfare Information Gateway 2020). CPS monitors families who are potentially harming their children and intervenes if they find evidence of physical violence, emotional violence, sexual violence, or neglect (Child Welfare Information Gateway 2020). Parental substance use while caretaking a child or infant warrants a CPS visit in some states (Child Welfare Information Gateway 2020). Substance use during pregnancy makes CPS intervention more complicated, as the child in question has not been born yet (Guidelines for Addressing Pregnancies and New Babies, Department of Human Services, Child Welface n.d). As cannabis consumption is legal (depending on the state) for people over 21 years of age, and there are no restrictions legally on maternal consumption during pregnancy, cannabis is now in a class similar to alcohol and nicotine consumption (Guidelines for Addressing Pregnancies and New Babies, Department of Human Services, Child Welface n.d). However, pregnant patients may avoid prenatal care appointments for fear that health care workers may notify CPS of their cannabis consumption (Guidelines for Addressing Pregnancies and New Babies, Department of Human Services, Child Welface n.d).

#### Differences in verbal screening and toxicological testing for cannabis metabolites allows room for racial and socioeconomic biases to harm patients

During pregnancy, CPS recommends providers encourage cessation of cannabis consumption, though CPS has no legal jurisdiction until birth (Marijuana Pregnancy and Breastfeeding Guidance for Colorado Healthcare Providers Prenatal Visits 2017). At labor and delivery, the two primary methods of testing for cannabis consumption are a verbal screen or a toxicological test (Marijuana Pregnancy and Breastfeeding Guidance for Colorado Healthcare Providers Prenatal Visits 2017). Screening is a verbal questionnaire, while toxicological testing is measuring THC and THC metabolites in a biological sample collected at birth, such as baby’s blood, baby or mother’s urine, or meconium or umbilical cord blood (Marijuana Pregnancy and Breastfeeding Guidance for Colorado Healthcare Providers Prenatal Visits 2017).

A positive verbal screen for cannabis consumption does not require an automatic CPS referral, but a referral is recommended when physicians are concerned about the patient’s substance use (2021 Colorado Code n.d ). However, CPS does not elaborate on what substance use would be concerning or at what cutoff physicians should decide that the infants welfare has been threatened (Guidelines for Addressing Pregnancies and New Babies,Department of Human Services, Child Welface n.d). The lack of definitions of what substance use is considered concerning allows room for physician bias in which patients get reported (Chasnoff et al. 1990), (Paltrow and Flavin 2013). Biases cause increased testing and reporting of cannabis use in pregnant patients of color and with lower socioeconomic status (Chasnoff et al. 1990), (Paltrow and Flavin 2013), (Roberts 1991), (Woliver 2002), (Killing 2016). Increased testing and CPS reporting combined with the higher rates of consumption in these groups (Ko et al. 2019) discourages some pregnant patients from visiting their doctors, negatively affecting their health and the health of their babies (Racial and ethnic disparities continue in pregnancy-related deaths 2019).

#### Online forums of pregnant and postpartum patients spread dangerous recommendations about the safety of cannabis use during pregnancy and methods to avoid detection by physicians

There are online communities of cannabis-consuming parents such as the #CannaMommy hashtag on Instagram and TikTok and the Facebook page “Pot smoking moms who cuss sometimes” who post threads, videos, blogs, and forums instructing other pro-cannabis parents about how to wade through the potential legal repercussions or CPS involvement (CannaMommy n.d).

### Research on the effect of fetal cannabis exposure on offspring is sparse

Despite increasing social and legal acceptance of cannabis consumption, and the public perception of medical benefits of CBD, the literature regarding the impact of THC, CBD, minor cannabinoids, and terpenes consumption on pregnancy and fetal development is lacking (ElSohly et al. 2016), (Fine et al. 2019). Additionally, the delineation between dose, method of consumption, and trimester of consumption is lacking (ElSohly et al. 2016), (Fine et al. 2019).

#### Clinical literature

Clinical studies are critical to understand the effects of in-utero substance exposure (ElSohly et al. 2016), (Fine et al. 2019). However, clinical studies have inherent ethical and logistic limitations (ElSohly et al. 2016), (Fine et al. 2019). Due to the inability to ethically randomize pregnant patients to consume cannabis or not, we rely on retrospective studies instead (ElSohly et al. 2016), (Fine et al. 2019). Retrospective studies have caveats including the inability to control for dosing, timing, or consumption of other drugs such as nicotine or alcohol (ElSohly et al. 2016), (Fine et al. 2019). Additionally, many retrospective studies do not collect any biological samples like blood or urine and rely on the memory and willingness of participants to answer questions accurately (ElSohly et al. 2016), (Fine et al. 2019). With the ambiguous legal and social framework of substance use during pregnancy, patients may also artificially reduce the true amount of usage to avoid judgment, shame, or legal consequences (Metz et al. 2019), (Young-Wolff et al. 2021). Studies of substance use that were published in the 1980s and 1990s must also be taken with a grain of salt, as THC concentrations in cannabis products has been steadily increasing, so results may be underrepresenting the impact that fetal THC exposure of contemporary products has on offspring development (ElSohly et al. 2016).

Retrospective clinical studies elucidate the impact of cannabis exposure on postnatal behavior, including an increase in impulsivity and hyperactivity, negative effects on memory, verbal outcome measurements, and attentional processes, as well as decreased abstract and visual reasoning in the exposed child (Chia-Shan et al. 2011). Additionally, cannabis exposure is associated with an increase in mid-childhood psychosis rates with higher risk in male offspring (Fine et al. 2019), increased rates of cannabis consumption once the child reaches adulthood (Sonon et al. 2015), and increased adolescent cigarette smoking and cannabis use with stronger effects in male offspring (Porath and Fried 2005).

In addition to the risks to behavior, gestational cannabis consumption also effects physiologic development and health outcomes (Chia-Shan et al. 2011), (Viveros and Marco 2015), (Jansson et al. 2018), (Hurd et al. 2019). Cannabis exposure increased risk of neonatal morbidity (Metz et al. 2017), including increased frequency of infection morbidity, as well as neurological morbidity (Metz et al. 2017). Cannabis-exposed 3–6-year-olds showed higher cortisol levels in their hair hormone analysis, greater anxiety, aggression, and hyperactivity in their behavioral analyses, and a reduction in the high-frequency component of heart rate variability at baseline meaning reduced vagal tone (Rompala et al. 2021). Consumption of cannabis products during pregnancy reduced expression of immune system genes including those of the type I interferon, neutrophil, and cytokine-signaling pathways in the placenta (Rompala et al. 2021). Further information on this data can be found in *comprehensive review papers *(Chia-Shan et al. 2011), (Viveros and Marco 2015), (Jansson et al. 2018), (Hurd et al. 2019). These physiologic impacts are critical, as they may alter health and development of the exposed baby over their childhood and adolescence (Chia-Shan et al. 2011), (Viveros and Marco 2015), (Jansson et al. 2018), (Hurd et al. 2019).

There is little to no published clinical data on how CBD, minor cannabinoids, or terpenes effect fetal development.

#### Preclinical literature

Preclinical literature is critical as it can answers questions that clinical studies ethically cannot. Most preclinical literature regarding gestational cannabis consumption involves dosing pregnant mice or rats with cannabinoids (Lee et al. 2021), (Dong et al. 2019), (Natale et al. 2020). Murine model research of fetal THC exposure highlights physiologic (CannaMommy (n.d)), (Lee et al. 2021), (Dong et al. 2019), (Natale et al. 2020), (Benevenuto et al. 2017), (Rubio et al. 1995), (Fish et al. 2019), (Frau et al. 2019), (Vela and Martín S, García-Gil L, Crespo JA, Ruiz-Gayo M, Fernández-Ruiz JJ, García-Lecumberri C, Pélaprat D, Fuentes JA, Ramos JA, Ambrosio E. 1998) and behavioral changes (ElSohly et al. 2016), (Manduca et al. 2020), (Bara et al. 2018) in offspring. Some findings show differential effects based on sex of the offspring (Frau et al. 2019), (Manduca et al. 2020), (Bara et al. 2018). THC is commonly consumed in combination with other widely available drugs such as nicotine, CBD, and alcohol (Forray et al. 2015). Murine research shows that fetal exposure to THC in combination with nicotine impacts THC metabolism (Breit et al. 2021), THC in combination with CBD altered offspring behavior and physiology (Maciel et al. n.d), (Kanyo et al. 2021), and craniofacial development (Fish et al. 2019). While the research investigating CBD exposure along during pregnancy is a new point of interest, the findings are moving (Ochiai et al. 2021), (Pandelides et al. 2020). Murine data show fetal CBD exposure alters offspring cognition and brain development (Swenson et al. 2022) and alters physiologic development (Pandelides et al. 2020) and reproductive ability (Pandelides et al. 2020).

There is little-to-no data regarding how exposure to minor cannabinoids and terpenes effect fetal development.

### Physicians lack training and guidance on the effects of cannabis consumption during pregnancy

#### Guidelines on medicinal cannabis use for obstetric physicians are recent

The American College of Obstetrics and Gynecology (ACOG) released guidelines for physicians regarding gestational cannabis consumption in 2017 (Mark et al. 2017). They recommend all physicians discuss consumption of tobacco, alcohol, cannabis, and other pharmaceutical and recreational drugs both before pregnancy and in early pregnancy (Marijuana use during pregnancy and lactation 2017). ACOG states that pregnant people who report cannabis use should be “counseled about concerns regarding potential adverse health consequences of continued use during pregnancy” and should encourage patients to diminish use (Marijuana use during pregnancy and lactation 2017). ACOG recommends physicians identify pregnant people who use cannabis for medicinal purposes and encourage the people to find alternative therapies for symptoms (Marijuana use during pregnancy and lactation 2017). ACOG also recognizes the lack of data regarding teratogenic effects of cannabis consumption during breastfeeding, and includes the statement that physicians should discourage use during that time (Marijuana use during pregnancy and lactation 2017).

#### Training on medicinal cannabis in medical schools is lacking

Despite medicinal cannabis being legal in the majority of American states, training in medical cannabis for physicians is lacking. Currently, there are no federally required formal trainings, though physicians may elect to learn medicinal cannabis information either informally through literature analysis or through formal classes (Cannabis Science and Medicine | CU School of Pharmacy n.d), (MS in Medical Cannabis Science Business n.d).

#### Medicinal cannabis training is available, though it requires physician monetary and time commitment

Training on medicinal cannabis is available but often not targeted or feasible for clinicians. One example in Colorado is the Cannabis Sciences Program at the University of Colorado Anschutz Medical Campus where physicians and pharmacists can elect to take specific trainings on medicinal cannabis (Cannabis Science and Medicine | CU School of Pharmacy n.d). These programs cover cannabis as a whole for all clinical populations, with a small emphasis on pregnant populations ( Cannabis Science and Medicine | CU School of Pharmacy n.d). Formal training programs vary from individual small continuing education courses to certificates, then bachelors and masters’ degrees (Cannabis Science and Medicine | CU School of Pharmacy n.d), (MS in Medical Cannabis Science Business n.d). Continuing education is available for many states from online platforms like the Medical Cannabis Institute (Catalog n.d). Certificates average 12 credits of coursework and are available from multiple institutions (Cannabis Curriculum n.d), (Medical Cannabis Science and Therapeutic Management, Post-Baccalaureate Certificate Saint Louis University n.d), (Cannabis Medicine and Certificate n.d), (Home n.d). Bachelors’ degrees or minors programs (Minor in Cannabis Studies - School of General Studies Graduate Education | Stockton University n.d), (Cannabis Studies and at SUNY Morrisville n.d), (MSU in Medicinal Plant and Chemistry n.d) and master’s degrees (MS in Medical Cannabis Science Business n.d), (MS in Medical Cannabis Science and Therapeutics n.d) that focus on medicinal cannabis include the cannabis studies minor or certificate are available at multiple institutions. While these programs allow for variability of training intensity, they also require physicians to commit to both monetary and time requirements of these programs, which is an unrealistic expectation for physicians given the already existing demands of the profession.

#### Medical schools recognize the discomfort of providers on the topic of cannabis

Medical school deans were interviewed about whether their curriculum would prepare trainees for prescribing medical cannabis or informing patients about it (Evanoff et al. 2017). Twenty-five percent of deans reported their graduates were “not at all” prepared to answer questions about medical cannabis, and 66.7% of deans reported their graduates were “not at all” prepared to prescribe it (Evanoff et al. 2017). Conversely, 24.0% of deans believed their graduates were “moderately, very, or extremely prepared” to answer cannabis questions, and only 6.0% believed graduates were equally qualified to prescribe it (Evanoff et al. 2017). Interestingly, 48.4% of deans either agreed or strongly agreed that formal education in medical cannabis should be included in *undergraduate* medical education (Evanoff et al. 2017). However, the issues surrounding medical cannabis are rarely available at undergraduate institutions, and medical students enter formal training with widely varying undergraduate degrees (Cannabis Science and Medicine | CU School of Pharmacy n.d), (MS in Medical Cannabis Science Business n.d). When interviewing medical residents and fellows, confidence levels held steady to those of the deans (Evanoff et al. 2017). When analyzing location of medical training, 82 of 145 medical schools (56.6%) were located in a state where medical cannabis was legal (35 states as of 2018), though only 9 (13%) had *any mention* of medical cannabis in their submitted curricula (Evanoff et al. 2017).

### Dispensary workers (budtenders) have no required medical training and can undermine patients’ trust in physicians

#### Training on physiology is not required for dispensary workers (budtenders)

Individuals who sell cannabis at dispensaries are called budtenders (Anna Boiko-Weyrauch 2016). Despite being this point of contact, dispensary staff are not mandated to complete any form of training regarding pharmacodynamics or physiology of their products, let alone in vulnerable populations like pregnant people (Haug et al. 2016). Of budtenders who report having any required trainings (55%), the majority revolved around business, customer service, safety and regulatory compliance (Haug et al. 2016). When describing their role as a budtender, responders explain that their primary job responsibilities include customer service (91%), stocking inventory (79%), ordering supplies or dealing with vendors/growers (67%), counseling patients (63%), record-keeping (63%), budgeting/finances/accounting (46%), and other responsibilities (25%) such as human resources, delivery, marketing, packaging products, and creating signage (Haug et al. 2016).

#### Budtenders provide medicinal cannabis recommendations to vulnerable patients

Despite 63% of respondents including medical counseling in their job title, and despite serving a median of 425 patients per week, dispensary staff are not mandated to have any knowledge regarding dosing, drug interactions, and other clinically relevant measures (Guidelines for Addressing Pregnancies and New Babies, Department of Human Services, Child Welface n.d). When specifically analyzing the medical counseling the budtenders report doing, 94% of responders say they provide “advice, guidance, or counsel” to patients. When questioned about the types of counsel, 88% discussed administration methods (e.g., oral versus inhalation), potential cannabis side effects (80%), benefits of cannabis for specific symptoms (74%), and other recommendations (22%), which included “natural remedies, travel/shipping legal advice, dosing guidelines, and ailment or disease-specific information” (Dickson et al. 2018).

#### Budtenders specifically recommend cannabis to pregnant patients

To understand how budtenders would counsel a woman in early pregnancy suffering from nausea, Dickson et al. performed a “mystery caller” study in which they contacted 400 registered dispensaries in Colorado and asked a set of pre-determined questions regarding pregnancy-related nausea (Dickson et al. 2018). Many trends became obvious. The response of the budtender was usually based on personal or secondhand experiences rather than research or clinical recommendations (Dickson et al. 2018). Sixty-five percent of budtender responders based their recommendations on personal opinion throughout the study (Dickson et al. 2018). Another clear trend was a lack of understanding of the effects of cannabinoids (Dickson et al. 2018). Responses ranged from firm statements that cannabis was safe for the developing baby and the mother during pregnancy to suggesting trying different doses (Dickson et al. 2018). In fact, 36% of budtenders blatantly stated cannabis use is safe in pregnancy, and 69% recommended cannabis products specifically for morning sickness (Dickson et al. 2018).

#### Budtenders recommend pregnant patients not seek medical advice from an obstetrician

One of the most alarming trends among budtenders is the discouragement of pregnant people from contacting their physician about questions about cannabis use (Dickson et al. 2018). While some budtenders encouraged the pregnant woman (see: researcher) to consult their health care provider, others expressed animosity and distrust of physicians (Dickson et al. 2018). When directly asked if the woman should speak to her physician, budtenders often responded with negative views of physicians, with instructions ranging from finding a physician who is pro-cannabis to avoiding all physicians because they are only “pushing pills” and that research is “propaganda” (Dickson et al. 2018). Some budtenders discussed how if physicians think cannabis is safe for cancer, then it must be safe in pregnancy as well (Dickson et al. 2018).

#### Federal or state mandates could curb how budtenders recommend cannabis to pregnant patients

In Canada, where recreational and medicinal cannabis consumption has been legalized for adults since 2018, restrictions on who can provide cannabis-related medical advice has been effective (Vastis et al. 2020). Individual provinces and territories each implemented their own mandatory training programs with the intent to regulate the information that dispensary patrons would receive (Vastis et al. 2020). Canadian budtenders recommended *against* the use of cannabis 93% of the time, with a sample size of 456 dispensaries (Vastis et al. 2020) compared to 69% of budtenders in Colorado recommending the use of cannabis products during pregnancy (Dickson et al. 2018). In the Canadian group, only 3.7% of budtenders based their recommendation of personal opinion, while 88.1% of budtenders references dispensary policy (Vastis et al. 2020). 89.9% of budtenders deferred the pregnant person to her health care provider without prompting, while an additional 9.6% deferred to the health care provider upon prompting (Vastis et al. 2020). This stark difference between American and Canadian budtenders is postulated to be from differences in dispensary regulation. In the USA, there is no oversight board or regulation that holds budtenders accountable for spreading misinformation to their customers. Additionally, no individual state with legalized recreational or medicinal cannabis has requirements regarding what medicinal information budtenders are allowed to recommend.

## Discussion and recommendations

The majority of people who consume cannabis products throughout their pregnancy are not doing so haphazardly (Ko et al. 2020), (Westfall et al. 2009). Overwhelmingly, people who consume cannabis during pregnancy are looking for a way to cope with the symptoms of their pregnancy in a way that is best for them and for their fetus (Ko et al. 2020), (Westfall et al. 2009). Instead, these patients are working in a complex legal and sociopolitical framework, where they turn to multiple sources for information, including physicians, pharmacists, dispensary employees (budtenders), the internet and social media, and legal bodies like child protective services. These results highlight the prevalence of cannabis consumption during pregnancy, the risk that cannabis exposure poses to a fetus, and identifies multiple intervention points that researchers, clinicians, public health professionals, dispensary workers, pharmacists, and legislatures can make meaningful impacts for the pregnant people in their care.

### How can researchers make a difference?

#### Recommendation 1: Researchers must investigate nuances of fetal cannabis exposure

Animal research is necessary to provide data about safety, adverse effects, risks, and dose determination via multiple administration methods of cannabis in pregnancy (Translational Science Spectrum n.d). Barriers to cannabis research include obtaining licensing for a schedule 1 substance, timeliness in obtaining the drug for research, and obtaining funding for cannabis research. The National Institute on Drug Abuse (NIDA) should publish a clear guide to beginning cannabis research, including the order of steps researchers must follow. These include obtaining funding for cannabis research, obtaining schedule 1 compliant storage for cannabis product, obtaining institutional approval for all animal work with cannabis product, scheduling site and storage inspections, and instructions on obtaining material from the NIDA drug supply program. Researchers should educate themselves on federal funding for this research, including the 2023 notice of special interest from NIDA on the effect of cannabis use and cannabinoids on the developing brain (Notice Number: NOT-DA-20-039). Researchers should familiarize themselves with non-federal funding sources focused on cannabis research, including state-based initiatives like the Institute for Cannabis Research.

#### Recommendation 2: Researchers should make their main findings available and accessible in lay language, translated into multiple languages, for patients who want to do their own research

While formal peer-reviewed publications are pertinent for researchers to disseminate their findings, it is also necessary for researchers to make their main findings available for non-academic audiences. There is a responsibility on the researcher to communicate their findings using lay-audience appropriate terminology and to put these findings in a larger context that lay audiences can understand. Additionally, these communications must be accessible, including by being available online, in multiple languages, and not behind a paywall. This allows for patients to obtain scientifically based evidence on the potential risks of cannabis consumption on their own terms.

### How can clinicians make a difference?

#### Recommendation 3: Physicians should initiate honest and truthful conversations with their pregnant patients regarding cannabis consumption

Physicians should begin initiating conversations regarding cannabis consumption at early prenatal visits. These conversations should be patient-focused, non-derogatory and should focus on answering patient questions and educating patients on the known risks to ensure they make informed decisions. Physicians should be honest about the potential legal consequences in their state and should be upfront about the possibility of toxicological testing at labor and delivery.

#### Recommendation 4: Pharmacists should inquire about medicinal or recreational cannabis use when discussing prescription medications with pregnant patients

For pharmacists to give informed healthcare advice, it is pertinent that they are aware of drug interactions. To know if patients are consuming medicinal or recreational cannabis, and to provide appropriate prescription guidance, it is necessary for pharmacists to ask pregnant patients outright about their consumption while they are checking the patient’s prescription medications.

### How can labor and delivery units make a difference?

#### Recommendation 5: Birthing hospitals should implement substance use screening that includes cannabis at prenatal visits, should screen cannabis positive patients for cannabis use disorder, and should refer positive patients to substance use treatment

While many birthing hospitals screen for substance use at labor and delivery, there are not federal or state requirements in all areas on what substances must be screened for. Hospitals should implement substance use screeners, which include marijuana consumption, into their electronic medical record akin to anxiety and depression screeners that many hospitals use. When a patient self-reports cannabis use in the screener, the EMR should flag the Cannabis Use Disorder Test (CUDIT-R), a validated screening tool for cannabis use disorder (Cannabis Use Disorder Test n.d). If a patient screens positive for cannabis use disorder, the EMR should flag for referral out to locally based substance use treatment services that are available for pregnant and postpartum people. Referral to substance use treatment is recommended by ACOG as an intervention for pregnant and postpartum patients with a substance use disorder (Marijuana use during pregnancy and lactation 2017), (Policy Priorities n.d). This screening to referral process should be implemented at all prenatal visits, as well as at labor and delivery. Additional providers of obstetric care, including midwives, doulas, and nurse practitioners, family medicine physicians, and birth centers should implement substance use screeners and compile contacts of local substance use treatment facilities to offer to patients who report cannabis use.

### How can medical oversight programs make a difference?

#### Recommendation 6: Medical, pharmaceutical, and nursing program curricula should be amended to include medicinal cannabis, as well as recreational cannabis consumption in vulnerable populations including pregnant people

As medicinal cannabis is increasingly available, and as research is released supporting the medicinal benefits of cannabis, healthcare professionals need to be educated on the benefits, risks, and potential drug interactions that cannabis has. This education should highlight vulnerable populations, including pregnant people, adolescents, and the elderly. In addition to formal medicinal cannabis, many patients self-medicate with recreationally available cannabis without the oversight of a healthcare provider. This elevates the need for healthcare professionals to be informed.

#### Recommendation 7: Medical, pharmaceutical, and nursing licensing boards should recommend cannabis-specific continuing education courses

As online continuing education classes are available and are relatively inexpensive, ranging from $100 to 300, licensing boards which require ongoing continuing education credits should provide their trainees with recommendations for cannabis-specific options. This will show institutional support for trainees to understand the complexities of cannabis in patient populations.

#### Recommendation 8: The American College of Obstetrics and Gynecology (ACOG) should provide physicians with educational training and scripts regarding cannabis use during pregnancy

As currently practicing physicians have no requirement for formal education on cannabis consumption, the ACOG should release informational material targeted for obstetricians that explain the current known risks of cannabis consumption during pregnancy. In addition to their guidelines that recommend physicians discourage cannabis consumption, the ACOG should release scripts that highlight common questions, dispel common myths, and address known risks in lay language. These scrips should include the physiologic, behavioral, and legal consequences of cannabis consumption during pregnancy. Trainings should be made available for obstetricians to gain experience discussing cannabis so they are confident in these interactions with their patients.

### How can governmental bodies make a difference?

#### Recommendation 9: Funding bodies should prioritize and support grant applications for researchers studying commonly consumed substances during pregnancy

For research to be completed, it must be funded. Federal, state, and independent funding bodies should increase funding availability for researchers who are studying the effects of fetal cannabis exposure, exposure to cannabis in combination with other recreational or prescription medications, and cannabis components like cannabidiol, minor cannabinoids, and terpenes.

#### Recommendation 10: State-level child protective services should make explicit reporting guidelines for pregnant patients who screen or test positive for cannabis at labor and delivery

By allowing providers to decide when cannabis consumption during pregnancy is “concerning” and when it is not, CPS is allowing for harmful physician biases to differentially impact birthing people of different races and socioeconomic statuses (Chasnoff et al. 1990), (Roberts 1991), (Woliver 2002), (Killing 2016). Each state’s CPS should release specific reporting guidelines for when providers should notify CPS of positive cannabis screens or tests. CPS should also release, in lay language, an explanation for pregnant patients of what can happen if they screen or test positive for cannabis during their pregnancy.

#### Recommendation 11: State governments should implement requirements for basic budtender training and limitations on the provision of medical advice

Canada legalized medicinal cannabis in 2018 (Vastis et al. 2020) and provided direct instructions for dispensary workers on risks to vulnerable groups like pregnant people (Canada n.d). State legislators should introduce similar legislation which would minimize the amount of untrained medical advice that pregnant patients obtain. Additionally, states can introduce legislation that requires minimum training for workers at licensed dispensaries that include brief physiologic training and highlight potential medical risks (including risks of consumption during pregnancy).

#### Recommendation 12: State governments should introduce requirements for “if pregnant or breastfeeding” labels on cannabis products

In states with legal recreational and/or medicinal cannabis, legislatures should introduce requirements for product labeling akin that include details of risks to the developing fetus, akin to Michigan’s labeling requirements.

#### Recommendation 13: The federal government should remove cannabis from schedule 1 drug classification

At minimum, cannabis scheduling should be demoted to a Schedule II drug, akin to clinically studied, medicinally beneficial drugs such as morphine, hydrocodone, and phenobarbital (Controlled Substance and Schedules n.d). Both Schedule 1 and Schedule 2 drug categorizations acknowledge the high potential for abuse, though Schedule 2 does not include the no medicinal evidence clause (Controlled Substance and Schedules n.d). This change in scheduling would dramatically decrease barriers to both preclinical and clinical research, where researchers would not have to pursue the complicated and drawn-out process of obtaining a DEA Schedule 1 license, sourcing from backlogged pharmaceutical companies, and broadening the topics of research that can be conducted. In tandem, rescheduling of cannabis will ease the ability for researchers to investigate cannabidiol.

### Strengths and limitations

Strengths of this review include the breadth and depth of the content covered, the comprehensive view of substance use during pregnancy from multiple perspectives, and the inclusion of studies and information from multiple facets of society. Limitations of this research includes the relatively limited availability of data focused specifically on cannabis consumption during pregnancy, the complexity of the sociopolitical field of substance use during pregnancy, and the differential methodologies of each study included herein.

## Conclusions

The legal and sociopolitical landscape that encompasses cannabis consumption in the USA is changing rapidly. Pregnant people consume cannabis products with little reliable research to inform them of the potential risks or benefits. For patients to make informed decisions regarding their pregnancies, it is necessary that we as researchers, healthcare providers, public health professionals, and governmental representatives work to address these major gaps. This narrative review serves to highlight alarming gaps in research availability, research restrictions, clinical management of gestational cannabis use, budtender recommendations, and healthcare worker training. This work exemplifies that cannabis consumption is increasing in a pregnant population, that pregnant patients fear legal and medical repercussions of reporting their consumption, and that there are multiple contact points with pregnant patients that could be better utilized to improve patient education. This work also highlights specific interventions that researchers, physicians, pharmacists, birthing hospitals, medical and pharmacy training programs, budtenders, the American College of Obstetrics and Gynecology, funding bodies, child protective services, and state and federal governments can implement in order to best serve and educate pregnant patients on the risks of cannabis use during pregnancy.

## Data Availability

Not applicable.

## Abbreviations

NVP: Nausea and vomiting in pregnancy
THC: Tetrahydrocannabinol
CBD: Cannabidiol
CPS: Child protective services
ACOG: American College of Obstetrics and Gynecology
NIDA: National Institute of Drug Abuse
